# Did the pandemic virus A(H1N1)PDM09 interfere other respiratory viruses? Evidence from the subtropical city Hong Kong

**DOI:** 10.1186/2047-2994-4-S1-P99

**Published:** 2015-06-16

**Authors:** L Yang, KH Chan, L Suen, CM Wong

**Affiliations:** 1 School of Nursing, The Hong Kong Polytechnic University, Hong Kong; 2 Department of Microbiology, The University of Hong Kong, Hong Kong; 3 School of Public Health, The University of Hong Kong, Hong Kong

## Introduction

The first wave of the novel influenza virus A(H1N1)pdm09 in the subtropical city Hong Kong coincided with the summer epidemics of seasonal influenza and other common respiratory viruses. We hypothesize that this virus could have interfered the regular seasonality of other respiratory viruses.

## Objectives

We obtained surveillance data from a tertiary hospital to test the hypothesis of viral interference.

## Methods

Weekly age-specific numbers of positive specimens for influenza A(H1N1)pdm09, seasonal A(H1N1), A(H3N2), influenza B, respiratory syncytial virus (RSV), adenovirus and parainfluenza were aggregated into the age groups of 0–4, 5–17, 18–64, and 65+ years during 2004 to 2013. Wavelet analysis was used to assess the temporal patterns of age-specific epidemic curves of other respiratory viruses were compared across the pre-pandemic, pandemic and post-pandemic periods. The epidemic peak time of each virus was also calculated separately for the winter and summer peaks in the pre- and post-pandemic seasons.

## Results

Positive proportions of viruses other than A(H1N1)pdm09 markedly decreased in all the age groups during the first pandemic wave. After the first wave of the pandemic, the positive proportion of A(H3N2) increased, but those of B and RSV remained slightly lower than their pre-pandemic proportions. Changes in seasonal pattern were also observed, but inconsistent across virus-age groups. As compared to the pre-pandemic period, the delayed peaks during the post-pandemic period were observed in A(H3N2) in all age groups and RSV of the 0-4 age group. By contrast, influenza B, adenovirus and parainfluenza showed slightly earlier winter and summer peaks in most age groups.

## Conclusion

There is some evidence that age distribution, seasonal pattern and peak time of other respiratory viruses have changed since the pandemic. These changes could be the result of viral interference, but could have also been caused by changing health-seeking behavior and other unknown mechanisms. The observed changes suggest a need to consider viral interference while formulating control policies, particularly the vaccination policy in different age groups.

## Disclosure of interest

None declared.

